# *AXEAP*: a software package for X-ray emission data analysis using unsupervised machine learning

**DOI:** 10.1107/S1600577522006786

**Published:** 2022-07-21

**Authors:** In-Hui Hwang, Mikhail A. Solovyev, Sang-Wook Han, Maria K. Y. Chan, John P. Hammonds, Steve M. Heald, Shelly D. Kelly, Nicholas Schwarz, Xiaoyi Zhang, Cheng-Jun Sun

**Affiliations:** a X-ray Science Division, Advanced Photon Source, Argonne National Laboratory, 9700 South Cass Avenue, Lemont, IL 60439, USA; b Department of Physics Education and Institute of Fusion Science, Jeonbuk National University, Jeonju 54896, Republic of Korea; c Center for Nanoscale Nanomaterials, Argonne National Laboratory, Argonne, IL 60439, USA; ESRF – The European Synchrotron, France

**Keywords:** AXEAP, XES, unsupervised machine learning, user-friendly interface

## Abstract

The *Argonne X-ray Emission Analysis Package* (*AXEAP*) has been developed to calibrate and process X-ray emission spectroscopy (XES) data collected with a two-dimensional (2D) position-sensitive detector. *AXEAP* is designed to convert a 2D XES image into an XES spectrum in real time using both calculations and unsupervised machine learning. *AXEAP* is capable of making this transformation at a rate similar to data collection, allowing real-time comparisons during data collection, reducing the amount of data stored from gigabyte-sized image files to kilobyte-sized text files. With a user-friendly interface, *AXEAP* includes data processing for non-resonant and resonant XES images from multiple edges and elements. *AXEAP* is written in *MATLAB* and can run on common operating systems, including Linux, Windows, and MacOS.

## Abstract 2

*AXEAP*, a program that can process high-resolution emission spectrum data quickly, has been developed based on machine-learning algorithms.

## 1. Introduction

X-ray absorption spectroscopy (XAS) and X-ray emission spectroscopy (XES) are two of the most common spectroscopic techniques used at synchrotron radiation facilities. In particular, both resonant and non-resonant XES have received significant attention in investigations of the electronic structure of materials, resulting in the development of a variety of X-ray emission spectrometers (Vankó *et al.*, 2006 ▸; Pollock *et al.*, 2014 ▸).

One class of such spectrometers is the miniature X-ray emission spectrometer (miniXES), which utilizes micro-focused beam with relatively short working distance between sample and analyzers. In a miniXES, flat analyzer crystals are arranged in a von Hamos or Johann geometry, and a position-sensitive detector (PSD) is used to detect the energy-resolved signal (Pacold *et al.*, 2012 ▸; Mattern *et al.*, 2012 ▸; Dickinson *et al.*, 2008 ▸; von Hámos, 1933 ▸). MiniXES-style spectrometers carry significant advantages in terms of signal strength, energy resolution, versatility, and ease of use in comparison with instruments based on spherically bent crystal analyzers (SBCA), making them an attractive option for a variety of XES beamlines across the world. MiniXES spectrometer capabilities have drawn interest from scientists from a variety of fields, such as biology, chemistry, and material science (Castillo *et al.*, 2021 ▸; Fransson *et al.*, 2018 ▸; Kucheryavy *et al.*, 2016 ▸). The current tools for working with XES data are limited and can only process one XES edge at a time; furthermore, the key process of defining regions of interest (ROIs), required to turn the image into a spectrum, is carried out manually, resulting in differences depending on personal preference and skills. Importantly, this manual process is slow and not capable of providing real-time information to the scientist. For these reasons, software development is warranted in order to process multiple XES edges and multiple images in real time with unbiased ROIs and with flexibility in the use of various PSDs.

To address these problems, we have developed a new XES analysis package, *Argonne X-ray Emission Analysis Package* (*AXEAP*), for processing emission data collected by a variety of two-dimensional (2D) PSDs based on unsupervised machine learning. Since the data collection method considers the intensity of pixels according to their vertical and horizontal positions, the data are usually saved as an image file. Mining meaningful data from an image can be tedious and time-consuming. As a result, many research groups have applied machine learning in the image data processing workflow (Alloghani *et al.*, 2020 ▸; Verbeeck *et al.*, 2020 ▸; Lieber *et al.*, 2013 ▸). Development of *AXEAP* has been directed by two important motivations – convenience and reliability of high-speed data processing.

The first motivation is to provide a user-friendly interface for data analysis, reduction, and comparison. Currently, it is difficult and time-consuming to verify the spectral form of XES during measurements. There is no commonly used software to convert the X-ray distribution on a 2D-PSD into spectral form. Manual ROI selection can be a daunting task for the non-expert. *AXEAP* was designed to incorporate a variety of features so that the task of transforming images into spectra is straightforward. In addition, the software includes a variety of features that allow data comparisons in real time and at the beamline during an experiment for the purposes of finding and correcting any issues that might arise, as well as experiment steering to make sure that the data collected are useful. This software package seeks to make the miniXES spectrometer, and accompanying XES and resonant XES (RXES) techniques, more accessible to the synchrotron user community.

The second motivation is to reduce data quickly from images to spectra in a reliable way so that the images do not need to be saved and transferred but can stay at beamlines for a while and then be deleted. The upcoming synchrotrons of the future are expected to revolutionize data collection time due to higher brightness and improved beamlines, which will allow the collection of vast amounts of data in the field of *operando* and time-resolved experiments. Therefore, a method to analyze vast amounts of data quickly and accurately is required. To accomplish these tasks, *AXEAP* provides functions for automatic setup, parallel calculation, and batch processing through a user-friendly interface.

## 2. Methodology of X-ray emission data acquisition

To understand how *AXEAP* works, we will first describe the methodology for collecting XES spectra through some simple examples. All data in this paper were collected at the 20-ID-C beamline of the Advanced Photon Source (APS) at Argonne National Laboratory, USA. Monochromatic X-rays, focused down to a diameter of 50 µm with an energy resolution of approximately 1 eV at the Ni edge and ∼0.8 eV at the Mn edge using a Si(111) double-crystal monochromator and Kirkpatrick–Baez (KB) mirrors, were calibrated to the peak of the first-derivative of the absorption edge for Mn, Co, and Ni *K*-edges from Mn, Co, and Ni foils, respectively.

**Figure 1:**
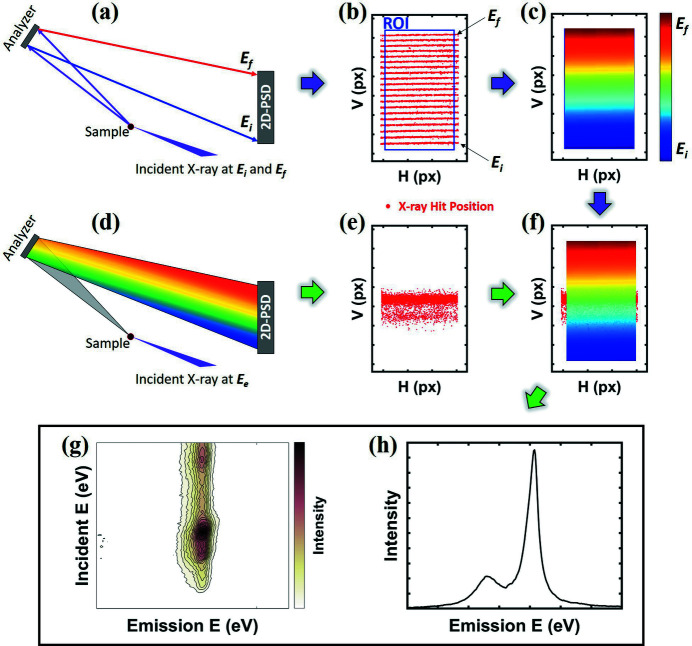
Workflow for obtaining a spectral form of the Mn *K*β_1,3_ emission of MnO. (*a*) Schematic of the optical geometry for obtaining elastically scattered X-rays with two different energies, *E*_i_ (6445 eV) and *E*_f_ (6540 eV). (*b*) Distribution of elastically scattered X-rays on the horizontal H (px) and vertical V (px) pixels of the 2D-PSD for an incident energy range of *E*_i_ and *E*_f_ at 5 eV intervals. The blue box indicates the ROI that is described in the text. (*c*) CEM corresponding to the energy distribution in the ROI of (*b*). (*d*) Schematic of the geometry used to obtain emission X-rays at a specific energy *E*_e_. (*e*) Distribution of emitted X-rays on the 2D-PSD. (*f*) Applying CEM to the distribution of (*e*). (*g*) Mn *K*β_1,3_ RXES and (*h*) Mn *K*β_1,3_ XES of MnO.

The process of XES data acquisition includes energy calibration and emission spectrum measurements. Fig. 1 ▸ illustrates the overall procedure for obtaining an emission spectrum from Mn(II) oxide. Most experimental designs for obtaining hard X-ray emission spectra with ∼1 eV resolution are based on a 2D-PSD, a micro-focused beam, and a spectrometer. In this approach, when the sample is exposed to incident X-rays, data acquisition is divided into two steps: distribution of elastic X-ray scattering for energy calibration, and distribution of inelastic emission X-rays.

Step 1. Obtaining the distribution of elastic X-ray scattering is a calibration process for using the 2D-PSD as an energy-dispersive detector. The target sample is exposed to monochromatic incident X-ray beam corresponding to energy from *E*_i_ to *E*_f_ at specific energy intervals. Since the incident angle of X-rays with a specific energy to a certain position of the analyzer is fixed, only elastic X-ray scattering that satisfies the Bragg condition are diffracted into the 2D-PSD [Fig. 1 ▸(*a*)]. Fig. 1 ▸(*b*) shows the distribution of elastic X-ray scattering positions of 20 exposures taken at 5 eV steps from 6445 eV to 6540 eV using a monochromatic X-ray beam. In this experiment, the distribution of the elastic X-ray scattering position is clustered into lines for each step. Each pixel of the 2D-PSD counts and records the number of photons which come into the pixel. After the calibration measurement, a ROI should be established around the X-ray hit positions to calibrate the data. Within this region, calibration is conducted using linear regression and pixel interpolation as shown in Fig. 1 ▸(*c*). In this paper, the defined relationship between incident X-ray energy and X-ray hit position is referred to as the calibration energy map (CEM).

Step 2. Obtaining the distribution of inelastic X-ray intensities is the process for collecting the emission spectrum from the sample. The choice of incident X-ray energy for obtaining an emission distribution is slightly different depending on the purpose of the measurement and can be either non-resonant (well above the absorption edge) or resonant (at or near the absorption edge). Since RXES is a two-photon coherent optical process, it combines a series of images taken using different incident X-ray energies. Whereas, in the non-resonant case, an emission distribution of X-rays is usually measured at least 150 eV above the absorption energy to avoid the interaction of valence-level electrons. When the sample is exposed to an incident beam that is either resonant or non-resonant, emission X-rays diffracted by the analyzer arrive at the 2D-PSD as shown in Fig. 1 ▸(*d*). In the case of non-resonant XES, most of the X-rays counted on the 2D-PSD surface will be emission signals as shown in Fig. 1 ▸(*e*). Once the CEM and emission images are prepared, they are combined such that the CEM defines the energy axis and the emission image defines the emission intensity as shown in Fig. 1 ▸(*f*). The converted spectral forms for RXES and XES are shown in Figs. 1 ▸(*g*) and 1(*h*), respectively.

## 3. Interface design

**Figure 2:**
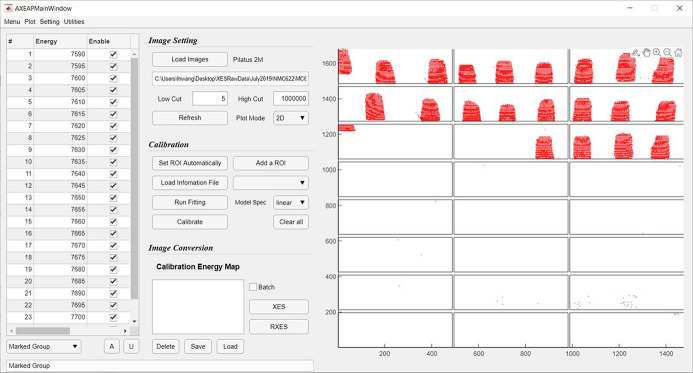
A screenshot of the main window of *AXEAP.* The graphic part on the right-hand side shows the distribution of the X-ray hit positions as red dots and is divided into 24 areas due to the specification of the detector used for measurement. The signal shown in the graphic part represents the 25 merged images as X-ray hit distributions, which were taken 25 times, each using different incident energies for Co *K*β_1,3_ emission line measurements. In this experiment, 22 InP (444) analyzers were used.

*AXEAP* consists of a main window with two sub-windows for image conversion. The main window is for image display and calibration processing of image data collected from 2D-PSDs. Most of 2D-PSDs store image data as 32-bit signed big tagged image file format (BigTIFF). This is because the 2D-PSD data acquisition system allocates one pixel as the signal collected from one diode in the detector and stores it in each pixel of the image data. The current *AXEAP* version is optimized for the 2D-PSD of DECTRIS, such as the Pilatus and Eiger series, but not limited to them. If different detectors are used for measurement, the option menu allows the user to manually define detector specifications such as the number of detector modules, total number of pixels, and dead zone between modules. Fig. 2 ▸ shows the main window of *AXEAP* when the calibration images are loaded. A table on the left side of the main window is prepared to control the loaded image data. Each image spectrum is represented by one row in the table. As described in the general methodology of the XES measurement, calibration images are measured in a series of scans. *AXEAP* can load multiple images at once with the X-ray energy imported by a separate scanning file, and users have options to display, hide and merge images using the control table. The ability to quickly inspect measured data allows users to flexibly process data.

When an image is imported into the program, based on the DECTRIS series, *AXEAP* automatically recognizes the detector type. The graphic part of the main window follows the set of detector specifications. For example, the graphic part in Fig. 2 ▸ is divided into 24 parts because the loaded images were measured with a Pilatus 2M detector, which contains 24 modules, each module consisting of 487 × 195 (width × height) silicon sensors. The sensor array is converted and stored in pixel units of the image. Between the modules, there is a dead zone that is 17 pixels high and 7 pixels wide, which is added to the image data. *AXEAP* employs 2D-scatter plots and 3D-mesh plots to visualize the pixel values.

In the center of the main window, there are options which allow the calibration process that invokes sub-windows, namely XES and RXES visualization windows. Each of the two sub-windows has its own graphic/plotting part to display the converted image in a similar manner to the main window. The sub-windows are designed to interact with the main window during data processing, providing feedback of each step in the data processing procedure. The resulting data can be saved and exported as text (.txt) and Excel (.xlsx) files.

*AXEAP* supports a function to convert multiple data that require the same procedure at once, via batch processing. By default, *AXEAP* is designed to process all images in one folder at once. However, if the emission data are sharing the same calibration process and are divided into multiple folders and saved, the batch process allows multiple folders to be recognized at once and processed in the same way. For example, if the sample is exposed to the incident beam for a long time during the XES measurement, the sample may be damaged. To prevent damage to the sample, multiple measurements are taken at multiple points in a short time to avoid overexposure of the beam at one point. In other words, several data groups will be created, but the data processing is the same. This is an example of minimizing beam damage, and time-resolved and *operando* experiments may also require large groups of data to go through the same process for different reasons. Therefore, this function can greatly streamline the data processing.

## 4. *AXEAP* features

### 4.1. Unsupervised machine learning technique: auto ROI setting

ROIs have two important functions: one is to separate stray signals from the main signal during data collection; the other is for identifying the data group. The shape of the ROI may be influenced by the experimental setup, but a rectangular shape is generally preferred over other shapes because it is very convenient to manually change the ROI size. *AXEAP* allows manual insertion of an ROI with a rectangular shape.

The selection of ROIs is typically performed manually and is one of the most tedious and time-consuming parts of processing XES spectral images. One or two ROIs settings can be completed in 1–2 min. However, if the spectrometer consists of multiple ROIs such as ten or more, the manual selection process, depending also on the experience of the individual user, may render it difficult to provide real-time data processing during the measurement. For example, Fig. 1 ▸(*b*) shows a set of calibration scans using one crystal measured in series scans, and the main window in Fig. 2 ▸ shows multiple groups with different crystals. To obtain spectral data from all calibration groups, as many ROIs as possible must be set as the number of data groups. In order to address this issue, *AXEAP* provides automatic ROI setting, based on the *K*-means clustering algorithm (Arthur & Vassilvitskii, 2007 ▸; Lloyd, 1982 ▸). The *K*-means clustering algorithm is used to cluster groups of data and is a type of unsupervised machine learning (UML) that helps rapid data processing. The software is designed to recognize X-ray hit positions on a 2D-PSD as data points, so it automatically divides the X-ray hit position by a user-defined number of clusters called *K*. *AXEAP* creates a data table according to the specifications of the defined detector and allows the user to enter *K* values for each module. After the user completes the *K*-value setting in the table, *AXEAP* automatically generates rectangular ROIs in seconds, in the following manner.

**Figure 3:**
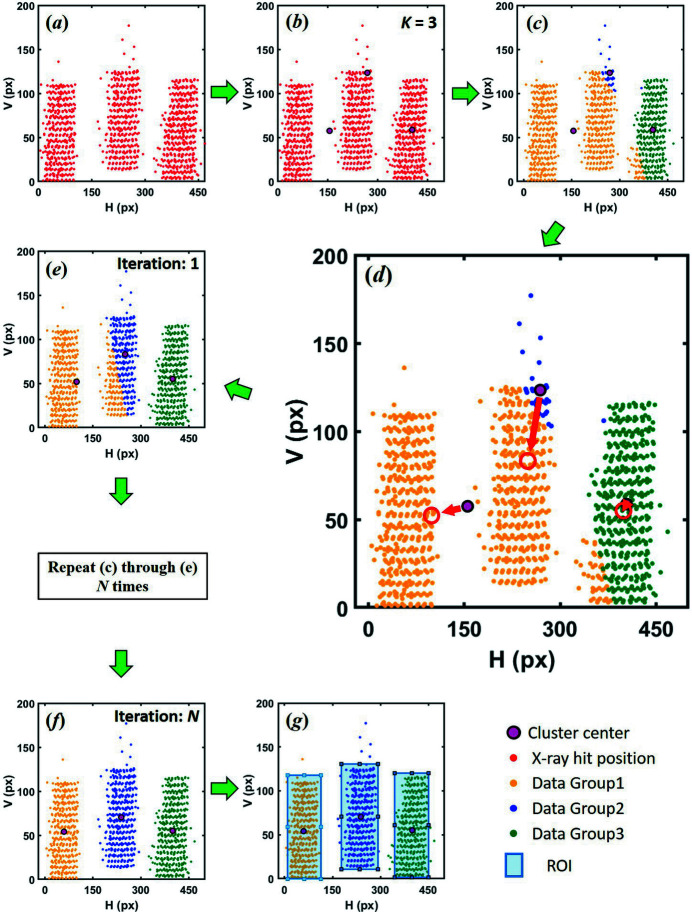
Workflow of automatic ROIs setting (automatic determination of ROIs). (*a*) Three clusters of merged X-rays hit positions with 17 linear distributions in one module of the 2D-PSD. The points have been reduced by 10 times to distinctly show the distribution. (*b*) Initially assigned three arbitrary centers of the clusters (black circles). (*c*) X-ray hit positions surrounding the cluster centers. (*d*) Reassigned locations of the three cluster centers. (*e*) Data clusters reassigned to new cluster centers. (*f*) Clusters and cluster centers are finally determined. (*g*) Three ROIs are automatically determined based on the clusters and their centers.

(1) Randomly generate cluster centers according to *K* values within a defined module size. For example, if users choose *K* = 3 in a module area, three cluster centers are generated as shown in Fig. 3 ▸(*b*).

(2) Compute the point-to-cluster-centroid distance of all X-ray hit points to each centroid, and then assign each X-ray hit point to the data group with the closest centroid [Fig. 3 ▸(*c*)].

(3) Compute the average of all the X-ray hit positions in each cluster to obtain new centroid locations. Based on this result, update new cluster centers [Fig. 3 ▸(*d*)].

(4) Repeat steps (2) through (3) until cluster assignments do not change, or the maximum number of iterations is reached [Figs. 3 ▸(*e*) and 3(*f*)].

(5) Determine the size of the ROIs based on the established centroid location [Fig. 3 ▸(*g*)].

Owing to the flat surface of the area detector and the potential non-perfect alignment of crystals, sometimes the image clusters are non-rectangular. In order to minimize the empty space of each ROI, we attempted to determine a standard method to determine the ROI size and shape based on the signal. We found that rectangular ROIs served the purpose well. Therefore, in the current version of *AXEAP*, we apply a rectangular ROI; furthermore, we also plan to develop the arbitrary shape of the ROI to meet the potential arbitrary shapes of calibration images for a broad user community.

**Figure 4:**
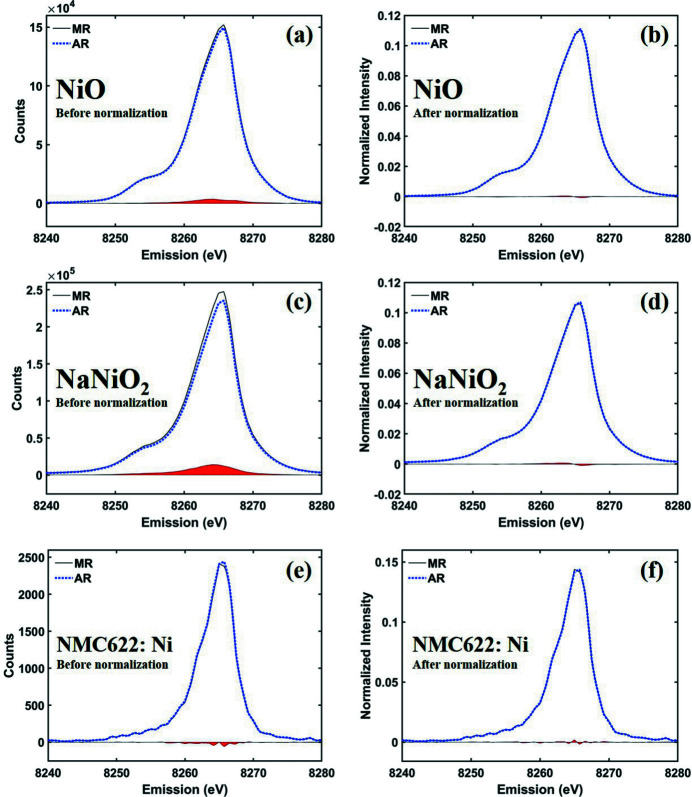
Ni *K*β_1,3_ spectra of nickel oxides converted by MR and AR. The solid black and the dotted blue lines represent MR and AR, respectively, for (*a*, *b*) NiO before and after normalization, (*c*, *d*) NaNiO_2_ before and after normalization, and (*e*, *f*) NMC622 at Ni *K*β_1,3_ emission spectra before and after normalization, respectively. The red colored areas indicate the differences between the MR and AR spectra.

**Figure 5:**
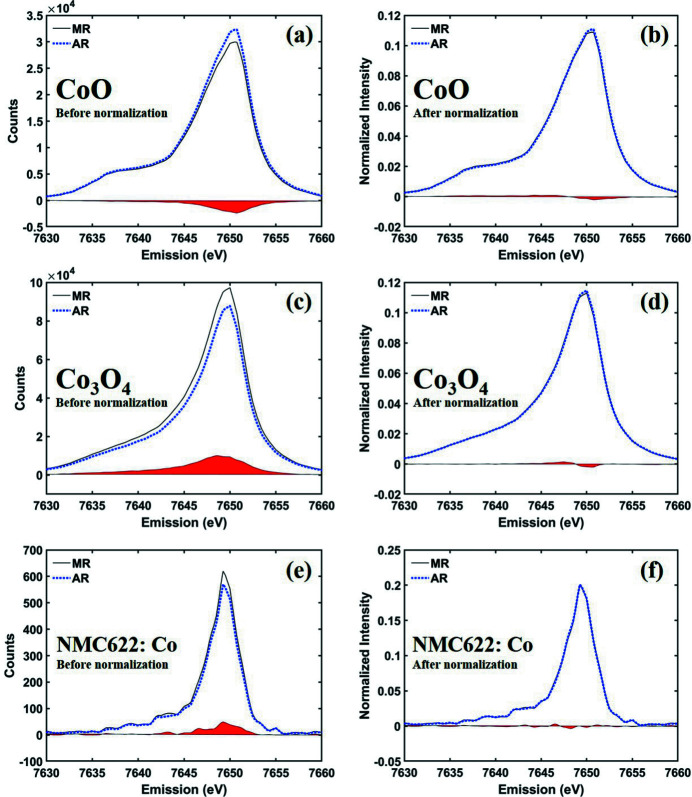
Co *K*β_1,3_ spectra of cobalt oxides converted by MR and AR. The solid black line and the dotted blue lines represents MR and AR, respectively, for (*a*, *b*) CoO before and after normalization, (*c*, *d*) Co_3_O_4_ before and after normalization, and (*e*, *f*) NMC622 at Co *K*β_1,3_ emission spectra before and after normalization, respectively. The red colored areas indicate the differences between the MR and AR spectra.

To evaluate the effectiveness of *K*-means clustering on the calibration images, we compared six measured XES data sets processed using manual ROI (MR) and automatic ROI (AR). Figs. 4 ▸ and 5 ▸ show spectra of several Ni and Co compounds, respectively, converted by merging multiple ROIs. The absolute intensity of the spectrum is greatly influenced by the ROI size due to the difference in signal counting within the effective area. In both ROI setting processes, there was a slight difference in the size and position of the ROIs, resulting in a difference in spectral intensity as shown in Figs. 4 ▸(*a*), 4(*c*), and 4(*e*) versus Figs. 5 ▸(*a*), 5(*c*), and 5(*e*), respectively.

In general, the absolute intensities of *K*β emission spectra are not used for analysis, but the relative intensity and ratio are considered important information (Pollock *et al.*, 2014 ▸). Since *K*β emission spectra are usually normalized to unit area for best comparison of relative intensities, we show the spectra normalized by integration area 1 in Figs. 4 ▸(*b*), 4(*d*), 4(*f*) and Figs. 5 ▸(*b*), 5(*d*), 5(*f*). In addition, we defined the integral of absolute values of the spectra difference ratio (IADR),

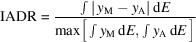

where *y*_M_ and *y*_A_ are the emission intensities converted to XES via the MR and AR setting, respectively. To define the spectral difference from the large signal, the denominator is divided by the larger integral value, *y*_M_ or *y*_A_. The calculation results are summarized in Table 1 ▸. Through this analysis, it is confirmed that, even if there is a large difference in AR and MR as shown in Fig. 5 ▸(*c*), the spectral difference significantly decreases after normalization [Fig. 5 ▸(*d*)]. This fact strongly implies that the AR setting does not significantly affect the spectral tendency, and is independent of the ratio AN/BN, as shown in Table 1 ▸. In other words, it proves that the ROI automatically set by *AXEAP* is effective for analysis and can dramatically reduce time, indicating that AR can be applied directly for data processing without user input.

If the data group is not clear between two or more groups, ROIs can overlap. For this reason, efforts are made to prevent overlapping of adjacent data groups during the alignment phase of the beamline experiment. Nevertheless, there are cases where overlapping is not prevented, and, as a result, clustering is not effective. *AXEAP* provides a function that allows users to freely change the ROI size and location after AR setting to correct for misclassified groups.

### 4.2. Calibration

Once the ROI is set up, the main window can calibrate the loaded images. This procedure determines an energy value corresponding to each pixel within the ROI displayed in the main window. As noted earlier, one image measured for calibration corresponds to a specific energy. Using the regression method, scattered distributions, as shown in Fig. 1 ▸(*b*), can be defined to specific energies, as a function of the locations of the horizontal and vertical pixels. The main window supports three types of regression models: constant, linear, and quadratic functions. The three regression models are designed to allow the user to flexibly define energy according to the shape of the X-ray hit distribution. The energy definition depends only on the X-ray hit position, regardless of the intensity, in order to respond appropriately if the intensity of the X-rays is weak. After the energy definition, pixels that are not defined within the ROI can be defined using interpolation and the CEM generation can be completed.

### 4.3. Emission image conversion: XES and RXES windows

**Figure 6:**
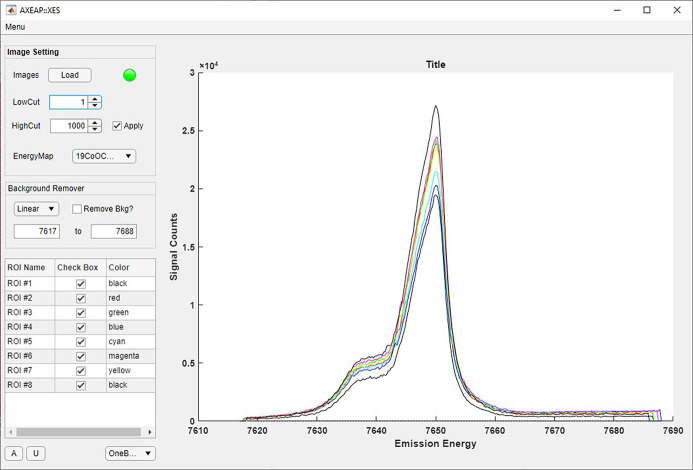
A screen shot of the XES window. The spectra show the Co *K*β_1,3_ XES of CoO for eight ROIs.

XES image data converted into spectral form are displayed in the XES window when a CEM corresponding to the loaded XES image data is present. Therefore, if at least one CEM is not prepared, the XES window is not activated. Once XES images are loaded into the XES window, the program begins loading the ROIs processed during calibration from a selected CEM and prepares a table containing the ROI name, check box, and plot line color, as shown in Fig. 6 ▸. At the same time, the image loaded from the XES window is output from the main window to help visualization, and plotted ROIs are named as in the prepared table. In addition, the XES window displays the transformed spectrum in the ROI displayed in the main window. The table located in the lower left of the XES window is designed to allow users to check the shape of the spectrum corresponding to each ROI. When users check or uncheck each ROI, the corresponding spectra are displayed or removed in the plotting part of the XES window.

**Figure 7:**
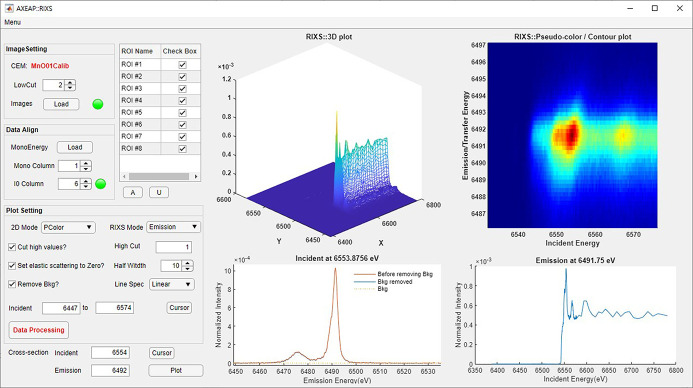
A screen shot of the RXES window with the four plots of XES (Mn *K*β_1,3_) and XANES (Mn *K*-edge) of MnO for eight ROIs. The 3D XANES image is rotatable.

As with XES image conversion, RXES image data conversion proceeds based on a CEM. Although the image conversion procedure is almost the same as for XES, RXES creates a three-dimensional spectrum, requiring more functional plotting options. To clearly visualize the three-dimensional data, the RXES window supports four plotting parts as shown in Fig. 7 ▸. One shows RXES on a three-dimensional mesh surface plot and another shows RXES on a contour/pseudo-color plot. Both plots represent the RXES spectrum, where the surface color varies depending on the *X*-axis, *Y*-axis, and intensity. An object plotted on the mesh axis can be rotated and zoomed. The other two axes are designed to show a cross section of the RXES spectrum plotted in mesh or contour/pseudo-color plot.

**Figure 8:**
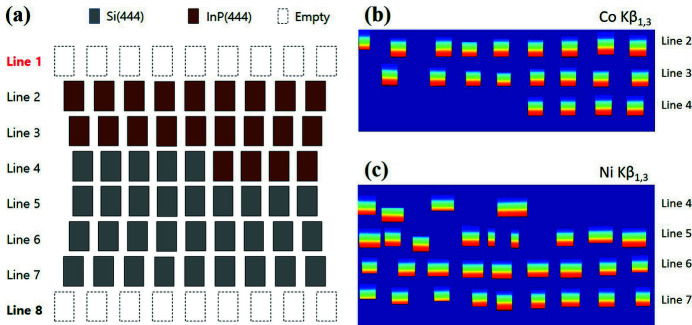
(*a*) Schematic of the spectrometer for multiple-edge measurement. Spectrometers are designed to accommodate up to 72 analyzers. Si(444) and InP(444) analyzers correspond to the energy range of Ni *K*β_1,3_ and Co *K*β_1,3_, respectively. (*b*) CEM for Co *K*β_1,3_. (*c*) CEM for Ni *K*β_1,3_.

One of the flexible functions in the XES and RXES windows is Multiple-edge Analysis, that converts a different region of emission in an image to spectral form. Fig. 8 ▸(*a*) shows the analyzer arrangement of the spectrometer for simultaneous measurements of Co *K*β emission and Ni *K*β emission, which was an example experimental scenario to investigate the spin and valence information of Co and Ni in Li-ion battery samples as a function of charge/discharge states. Therefore, two-element CEMs are required to convert an image into two types of spectra, as shown in Figs. 8 ▸(*b*) and 8(*c*). The Multiple-edge Analysis function can convert multiple-edge images into spectra using just a simple click if CEMs corresponding to each emission line are prepared. The spectra shown in Figs. 4 ▸(*e*) and 5 ▸(*e*) are results of the Multiple-edge Analysis function.

### 4.4. Parallel calculation and programming environment

RXES conversion can take longer than XES conversion when faced with a situation where a large amount of RXES image conversion is required. Optionally, users can enable parallel computation to solve this problem. When this function is activated, *AXEAP* counts the number of cores on the executing computer, then accelerates the image conversion procedure by multi-threading. As the processes are trivially parallel, the work speed increases almost linearly with the number of cores. For example, it takes 46 s to convert 260 images measured by a Pilatus 2M detector to RXES data using an Intel i7 tenth-generation single core; the software takes 8.9 s to complete the same RXES data processing, if users activate parallel mode in *AXEAP* to increase the number of CPU cores used from one to eight. In summary, parallel mode can shorten the RXES calculation time as the number of cores increases. The calculation time is displayed in the status text box of the main window.

*AXEAP* works on most common computer operating systems, including Linux, Windows, and MacOS. *AXEAP* is a free program, available by email request sent to the corresponding author. In order to run the program, *MATLAB Runtime 2021b* is required, a standalone set of shared libraries that enables the execution of compiled *MATLAB* applications or component without license. *MATLAB Runtime* can be accessed via the MathWorks website (https://www.mathworks.com/products/compiler/mcr/index.html).

## 5. Conclusion and future development

In this paper, *AXEAP* is introduced as a program focused on converting 2D X-ray emission spectroscopy image data into a 1D spectral form. *AXEAP* demonstrates similar performance to human labeling when unsupervised machine learning is activated. Other features are also designed to enable fast data processing, such as parallel execution and multiple-edge analysis. These outstanding functions enable rapid processing of vast amounts of data and helps the user analyze them easily.

Further functionalities integrating XES/RXES simulations will enhance the data analysis capabilities and are in development. In order to achieve real-time experimental steering, we are developing a user interface to incorporate *AXEAP* with the beamline data acquisition system, to stream multiple XES in real time. Real-time analysis will allow users to immediately respond to any errors or unexpected results that may occur during measurement, which helps in adapting experimental conditions and scientific discovery.

## Figures and Tables

**Table 1:** Sample list for comparison between MR and AR

		Integral of the absolute values of the spectra difference ratio (IADR)		
Sample name	No. of ROIs	Before normalization (BN) ×10^−2^	After normalization (AN) ×10^−2^	BN/AN
NiO	28	2.28	0.37	6.16
NaNiO_2_	28	5.78	0.62	9.32
NMC622:Ni	22	1.42	0.75	1.89
CoO	20	5.65	1.68	3.57
Co_3_O_4_	21	11.11	1.02	10.89
NMC622:Co	21	7.96	1.94	4.10
